# Gamma-irradiated rotavirus: A possible whole virus inactivated vaccine

**DOI:** 10.1371/journal.pone.0198182

**Published:** 2018-06-07

**Authors:** Shabihah Shahrudin, Cheng Chen, Shannon C. David, Eve V. Singleton, Justin Davies, Carl D. Kirkwood, Timothy R. Hirst, Michael Beard, Mohammed Alsharifi

**Affiliations:** 1 Research Centre for Infectious Diseases, Department of Molecular and Biomedical Science, School of Biological Sciences, University of Adelaide, Adelaide, SA, Australia; 2 Australian Nuclear Science and Technology Organisation, Lucas Heights, NSW, Australia; 3 Enteric Virus Group, Murdoch Childrens Research Institute, Parkville, VIC, Australia; 4 Gamma Vaccines Pty Ltd, Mountbatten Park, Yarralumla, ACT, Australia; University of South Florida St Petersburg, UNITED STATES

## Abstract

Rotavirus (RV) causes significant morbidity and mortality in developing countries, where children and infants are highly susceptible to severe disease symptoms. While live attenuated vaccines are available, reduced vaccine efficacy in developing countries illustrates the need for highly immunogenic alternative vaccines. Here, we studied the possible inactivation of RV using gamma(γ)-irradiation, and assessed the sterility and immunogenicity of γ-irradiated RV (γ-RV) as a novel vaccine candidate. Interestingly, the inactivation curve of RV did not show a log-linear regression following exposure to increased doses of γ-rays, and consequently the radiation dose required to achieve the internationally accepted Sterility Assurance Level could not be calculated. Nonetheless, we performed sterility testing based on serial passages of γ-RV, and our data clearly illustrate the lack of infectivity of γ-RV preparations irradiated with 50 kGy. In addition, we tested the immunogenicity of 50 kGy γ-RV in mice and our data illustrate the induction of strong RV-specific neutralising antibody responses following administration of γ-RV without using adjuvant. Therefore, whilst γ-RV may not constitute a replacement for current RV vaccines, this study represents a proof-of-concept that γ-irradiation can be applied to inactivate RV for vaccine purposes. Further investigation will be required to address whether γ-irradiation can be applied to improve safety and efficacy of existing live attenuated vaccines.

## Introduction

Rotavirus is an enteric pathogen that causes considerable morbidity and mortality, particularly in infants and children living in poor health conditions. Each year, RV infection causes over 100 million cases of gastroenteritis, and more than 200,000 deaths [1]. Approximately 80% of these deaths are children living in low-income countries [2]. RV infects mature enterocytes of villi in the small-intestine [3], causing clinical symptoms of nausea, vomiting, fever and diarrhea [4–6]. Severe symptoms of RV infection include malabsorption of nutrients, prolonged dehydration, and intussusception (IS), whereby one segment of the intestine becomes invaginated, causing inflammation, loss of blood flow, and significant pain [7].

Currently, there are 2 licensed oral RV vaccines: a single-strain vaccine, Rotarix® (GlaxoSmithKline Biologicals) and a pentavalent vaccine, Rotateq® (Merck & Co) [8]. Both provide protection against RV infection and RV-related gastroenteritis, but reduced efficacy has been reported in developing countries, where the burden of severe rotavirus disease is greatest [9]. Prior studies and clinical trials performed in infants in high-income settings, such as the United States and Europe, demonstrated RV vaccine efficacy exceeding 90% [10–12]. In contrast, reduced vaccine efficacy and protection rates have been reported in low-income settings such as Asia and sub-Saharan Africa [13–15]. This loss of efficacy may be related to host population differences and/or the high incidence of existing infections within the gut at the time of oral RV vaccination [16], which are thought to interfere with and potentially overwhelm the immune response to the RV vaccine [17]. Additionally, live attenuated vaccines have been associated with multiple clinical complications. For example, vaccine (RotaTeq®)-derived rotavirus has been reported to be transmitted from a vaccinated infant to an older, unvaccinated sibling, resulting in symptomatic RV infection with gastroenteritis and requirement for emergency medical care [18]. Moreover, an increased risk of IS has been associated with live attenuated RV vaccines, such as the RotaShield® vaccine, which was licensed in the United States in 1998 but withdrawn from the market within 9 months [7, 19]. Contemporaneous introduction of RV1 [Rotarix; GSK] and RV5 [RotaTeq; Merck] vaccines in Australia enabled a population-based assessment of the risk of IS, and illustrated the increased risk of IS after administration of both vaccines [20]. Furthermore, in many countries, the first dose of live attenuated rotavirus vaccine is administered around 2 months of age. This usually occurs before any possible diagnosis of severe combined immunodeficiency (SCID) disease in infants. Although it is rare, infection of SCID infants with the RV vaccine strain can lead to severe complications and hospitalisation due to their inability to generate protective immune responses [21, 22]. Overall, development of a safer and highly effective RV vaccine is an important step to better control RV infection.

Considering these issues, alternative RV vaccine approaches continue to be developed. These include an inactivated vaccine [23, 24], virus-like particle vaccines [25, 26] and recombinant subunit vaccines [27, 28]. In the current study, we tested the possibility of using γ-irradiation to inactivate whole RV for vaccine purposes. This approach allows abrogation of RV infectivity for enhanced safety, while maintaining immunogenicity to mimic that of live virus. Gamma-irradiation has been widely used to inactivate highly infectious agents for biochemical analysis, including Ebola, Marburg and Lassa viruses [29]. In addition, previous studies have illustrated the high immunogenicity of γ-irradiated vaccines, such as the γ-irradiated influenza A virus vaccine [30, 31] and the γ-irradiated pneumococcal vaccine [32]. We have previously proposed that γ-irradiated pathogens may be used to replace present day vaccines, particularly those associated with complications [33]. Considering the health risks associated with current RV vaccines and their reduced efficacies in Asia and sub-Sahara Africa, using γ-irradiation to inactivate RV for vaccine purposes is a promising alternative. Here, we show the inactivation curve of RV following exposure to increasing doses of γ-irradiation, and demonstrate the ability of γ-RV to induce strong RV-specific neutralizing Ab responses.

## Material and methods

### Ethics statement

This study was conducted in strict accordance with Australian Code of Practice for Care and Use of Animals for Scientific Purposes (7th edition [2004], 8th edition [2013]) and South Australian Animal Welfare Act 1985. Experimental protocols approved by Animal Ethics Committee at The University of Adelaide (Ethics number S/2014/107).

### Cells & viruses

African green monkey kidney epithelial cells (Vero and MA104) were grown and maintained in Dulbecco’s Modified Eagle’s Medium (DMEM, Gibco), + 10% FCS, 1% 2mM L-glutamine, and 1% penicillin/streptomycin. For MA104 cells, 0.5% 200mM sodium pyruvate was also added. Cells were kept at 37°C in a humidified atmosphere + 5% CO_2_. Vero cells were obtained from Prof Arno Mullbacher (The John Curtin School of Medical Research, ACT/Australia) and MA104 cells were provided by Dr Carl D. Kirkwood (Murdoch Childrens Research Institute, VIC/Australia).

Rhesus Rotavirus strain Rh452 (RV) was provided by Dr Carl D. Kirkwood (Murdoch Childrens Research Institute, VIC/Australia) and Semliki Forest Virus (SFV, avirulent A7 strain) was originally obtained from Prof Arno Mullbacher (The John Curtin School of Medical Research, ACT/Australia). RV and SFV were grown *in vitro* by infecting MA104 and Vero cells, respectively. RV was first activated with 10μg/mL trypsin (Sigma, T0303) at 37°C for 1 hour. Cells were infected at a multiplicity of infection (MOI) of 0.1, and infected flasks were incubated for 24 h at 37°C in a humidified atmosphere with 5% CO_2_. Culture supernatants were pooled, and clarified to remove cellular debris by centrifugation at 1500 rpm for 5 minutes. Clarified supernatants were stored at -80°C. RV infectivity was confirmed by fluorescent focus assay, and stock titre was estimated to be 3.6 × 10^7^ fluorescent focus units (FFU)/mL. In contrast, SFV infectivity was confirmed by plaque forming assay, and stock titre estimated to be 3 × 10^8^ plaque forming units (PFU)/mL.

### Fluorescent focus assay (FFA)

96-well tissue culture plates were seeded with 6 × 10^4^ MA104 cells/well and incubated for 24h. RV stock was activated by incubation with 10 μg/mL TPCK-trypsin (Sigma-Aldrich) in serum-free DMEM at 37°C for 1 h. Activated RV stock was then 10-fold serially diluted. 100 μL of diluted virus was added to MA104 monolayers. Plates were incubated for 30 minutes at 37°C to allow virus attachment to cells. An additional 100 μl of serum-free DMEM + 1μg/mL TPCK-trypsin was added to all wells, and plates were incubated for 18 h at 37°C. After incubation, monolayers were washed, then fixed and permeabilised with ice-cold acetone:methanol mixture (1:1 ratio) at 4°C for 15 min. Polyclonal mouse anti-RV sera was added to all wells and plates were incubated for 1 h at 4°C. After that, secondary Alexa Fluor® 488 goat anti-mouse IgG (Life Technologies, USA) was added and plates were incubated for 1 h at 4°C. Following incubation, plates were washed and cells were stained with DAPI (1 μg/mL, Sigma) for 30 min at RT. Images were acquired using a Nikon TiE inverted fluorescence microscope, and analysed using NIS elements software (Tokyo, Japan).

### Plaque forming assay (PFA)

SFV titres were estimated by plaque formation on semi-confluent monolayers of Vero cells as previously described [34, 35]. Samples were serially diluted on ice in PBS, and cells inoculated in duplicate with 0.1 mL aliquots of the diluted samples. Adsorption was for 1 h at 37°C followed by the addition of an agar overlay medium (0.9%). After 72 h incubation at 37°C, cells were fixed with 5% paraformaldehyde (BDH chemicals, Aust.) for 1 h. Overlays were removed, and fixed cells were stained with 0.2% crystal violet in ddH_2_O. The stain was washed and plaques enumerated using a light microscope.

### Vaccine preparations

RV and SFV samples were exposed to γ-irradiation (5–50 kGy) from a ^60^Co source at Australian Nuclear Science and Technology Organisation (ANSTO), NSW. Virus stocks were kept frozen on dry ice during transportation and irradiation. Irradiated samples were tested for reduction in titre using FFA for RV and PFA for SFV. In addition, the sterility of 50 kGy-irradiated RV was tested using 3 passages in MA104 cells. Cell monolayers were treated with trypsin-activated live RV or γ-RV using 4 x 10^5^ FFU-equivalent/well. Monolayers were incubated for 24 h, and culture supernatants were collected and used to infect new MA104 cell monolayers. Previously infected monolayers were stained for RV infection as per FFA. Lack of detectable FFU for all 3 passages in cells treated with γ-RV would indicate complete loss of infectivity following exposure to γ-irradiation. In addition, to illustrate the sensitivity of the 3 passages-based sterility testing, cell monolayers were infected with serially diluted trypsin-activated live RV (0.2 to 2000 FFU/well). Monolayers were incubated for 24 h, and culture supernatants were collected and used to infect new MA104 cell monolayers. Monolayers for all 3 passages were stained for RV infection as per FFA.

### Mice & treatment

Six to eight week old female C57BL/6 mice were supplied by Laboratory Animal Services, University of Adelaide, Australia. Using 3 mice/group, mice were primed twice intravenously (i.v.) on Days 0 and 14 with live or γ-irradiated RV (2 x 10^6^ FFU equivalent/mouse). Blood samples collected from all mice via submandibular bleeding 21 days post 2^nd^ vaccination.

### Measurement of RV-specific antibody responses

Levels of RV-specific total IgG, IgG1, and IgG2c in serum samples from immunised and control mice were determined by direct ELISA as described previously [36]. In brief, maxisorp plates were coated with Rh452 viral antigen diluted in bicarbonate coating buffer (Na_2_CO_3_, NaHCO_3_, in water, pH 9.6) and incubated overnight at room temperature. Non-specific protein binding sites were then blocked with PBS containing 2% skim milk powder for 2 h at room temperature. 50μL of serially diluted serum samples were added to the appropriate wells for 2 h at room temperature, followed by the addition of horseradish peroxidase conjugated goat anti-mouse IgG (Thermo Scientific), rabbit anti-mouse IgG1 (Invitrogen USA), or goat anti-mouse IgG2c (Southern Biotech USA). Plates were then incubated at room temperature for 2 h, and developed using TMB peroxidase substrate in the dark for 30 mins. The reaction was stopped with 2 mol H_2_SO_4_, and absorbance measured at 450/620 nm using a Microplate ELISA reader (Biotrack II plate reader). End-point titres expressed as the reciprocal of the last dilution where OD value ≥ cut-off value. Cut-off determined as mean + (3 × S.D.) of OD values of samples from control mice.

### In vitro neutralisation assay

96-well tissue-culture plates were seeded with 6 × 10^4^ MA104 cells/well. RV was activated by treatment with 10 μg/mL TPCK-trypsin (Sigma-Aldrich). Heat-inactivated (56°C, 30 min) serum samples were serially diluted, mixed with activated RV at a ratio of 1:1, and incubated for 1 h at 37°C to allow binding. 100 μL of each mixture was then added to MA104 cell monolayers at MOI of 0.005 and incubated for 18 h at 37°C. Cells were fixed, stained and visualised as described for FFA.

### Statistical analysis

Quantitative results expressed as mean ± SEM. Unpaired Student’s t-test used for comparison of data from two separate groups, and One-way ANOVA used for comparison of data from 3 or more groups. Statistical analysis performed using GraphPad Prism 6, version 6.0d (GraphPad Software, La Jolla, CA, USA). *P* values <0.05 (95% confidence) considered statistically significant.

## Results

### Inactivation curve of RV

**Fig 1:**
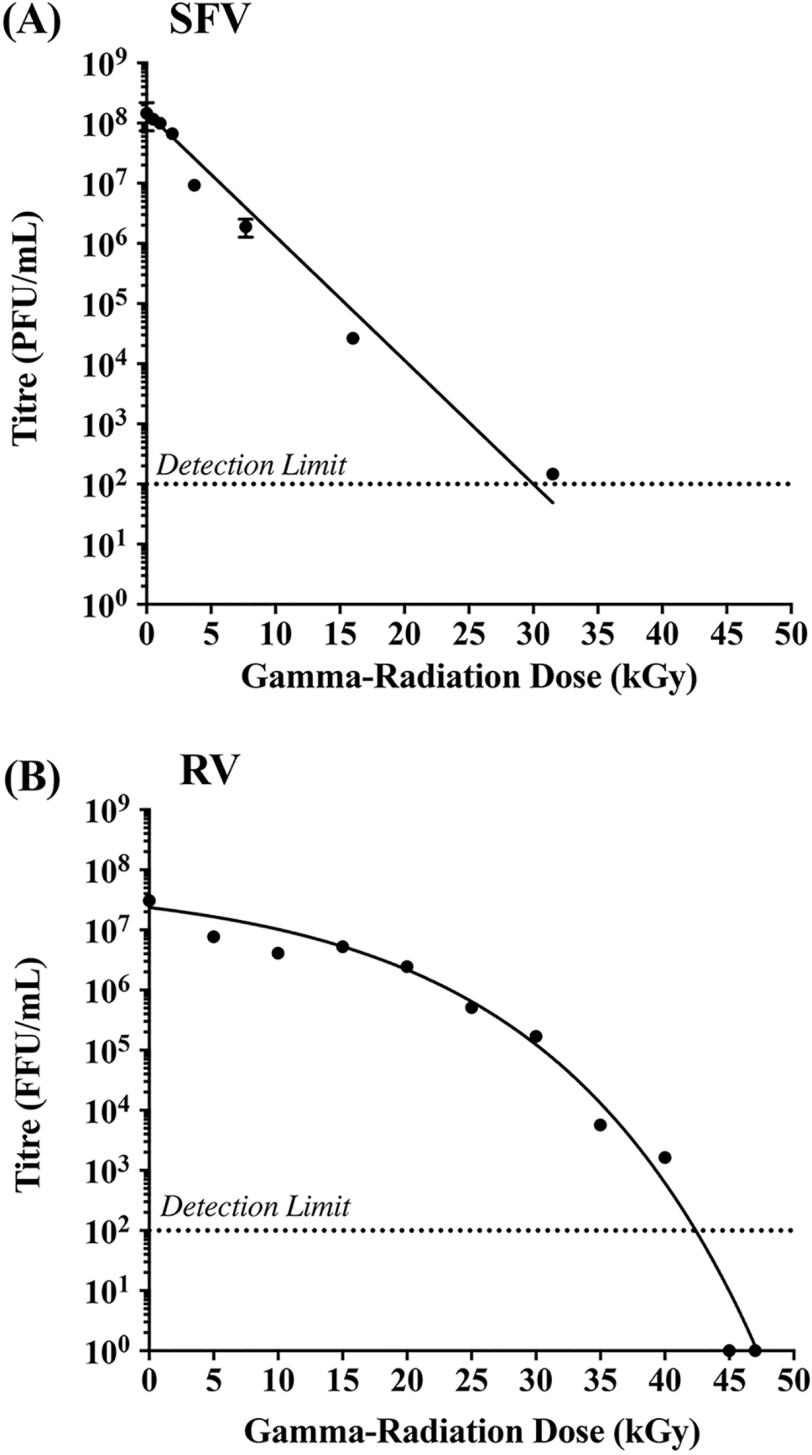
Inactivation curves of RV and SFV following exposure to γ-irradiation. SFV and RV samples were exposed to increased doses of γ-irradiation on dry-ice, and the reduction in virus titre was determined by (A) plaque forming assay for SFV, or (B) fluorescent focus assay for RV. All samples tested in triplicate and data presented as mean ± SEM.

To determine whether γ-irradiation can be used to inactivate RV for vaccine purposes despite the complex genome structure, we compared the inactivation curves of RV (11 segments of dsRNA genome) and SFV (single linear ssRNA genome). SFV and RV samples were exposed to increasing doses of γ-irradiation (5–50 kGy), and the effect of irradiation on virus infectivity was determined using FFA for RV and PFA for SFV. In general, inactivation curves can be used to estimate the D_10_ value and the Sterility Assurance Level (SAL) for a given pathogen. The D_10_ value indicates the radiation dose required to reduce viable virus tire by 90% (1-log reduction), whilst the SAL refers to the total dose required to achieve a theoretical titre of 10^−3^ or 10^−6^ units/mL. As shown in Fig 1A, no virus infectivity was detected in SFV samples exposed to irradiation doses above 30 kGy. In addition, the inactivation curve of SFV showed a distinct log-linear relationship between the increase in γ-irradiation dose and the associated loss of virus titre. In contrast, we did not detect a linear inactivation curve for RV (Fig 1B), and consequently the D_10_ value and SAL for γ-irradiated RV preparations could not be calculated. Nonetheless, we did not detect any infectivity by FFA for RV samples exposed to irradiation doses of 45 and 50 kGy.

### Sterility testing of 50 kGy γ-RV

**Fig 2:**
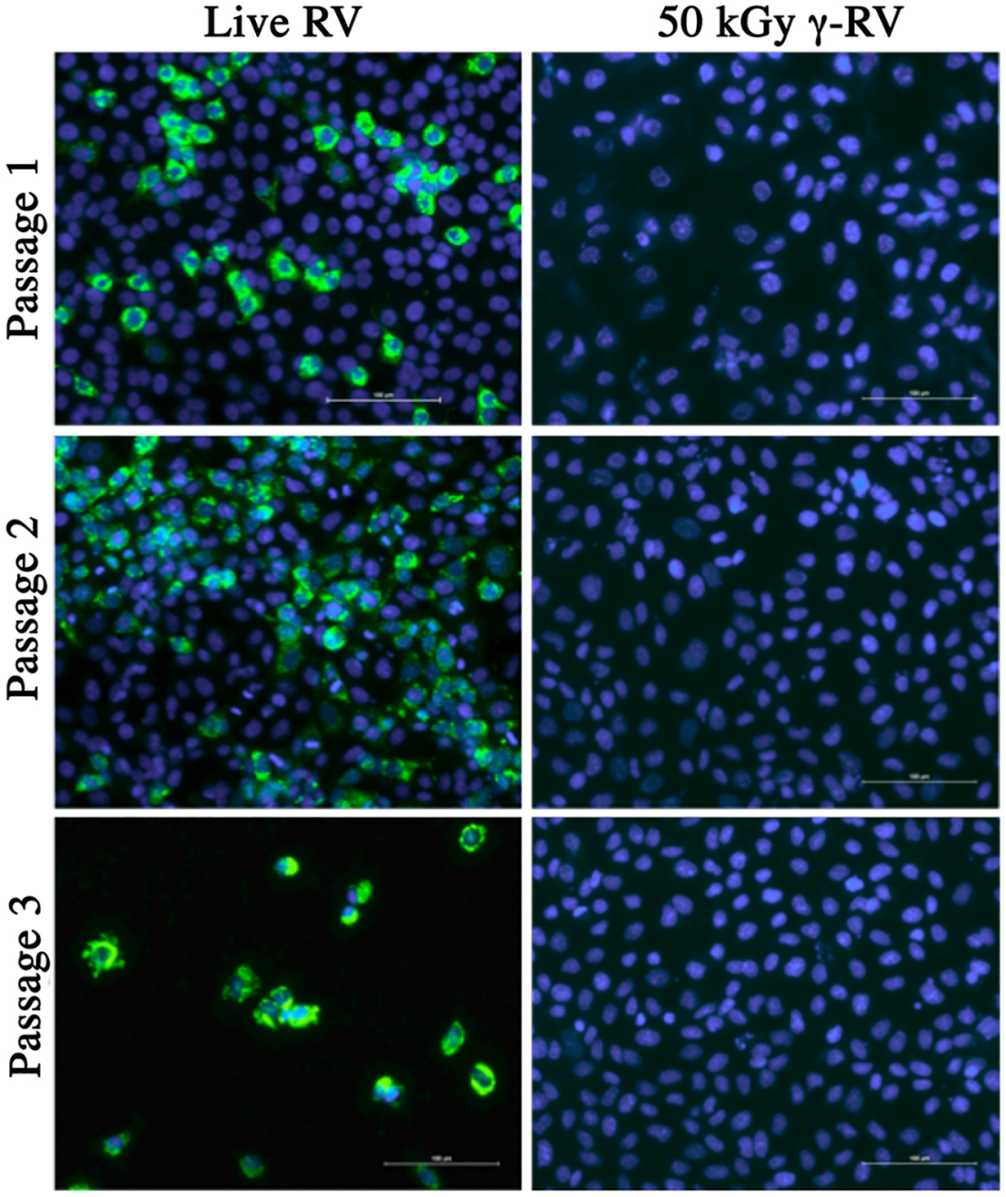
Sterility testing of 50 kGy γ-irradiated RV. Monolayers of MA104 cells were incubated for 24h with live RV or γ-RV at 4 x 10^5^ FFU-equivalent/well. Culture supernatant was harvested and used to infect new MA104 cell monolayers, and previously infected monolayers stained for RV infection by FFA. DAPI channel (blue) indicates cell nuclei, and FITC channel (green) indicates RV infection. Stained monolayers visualised using Nikon TiE inverted fluorescence microscope. Scale bar = 100 μm. Images representative of 5 replicates per sample for each passage.

**Fig 3:**
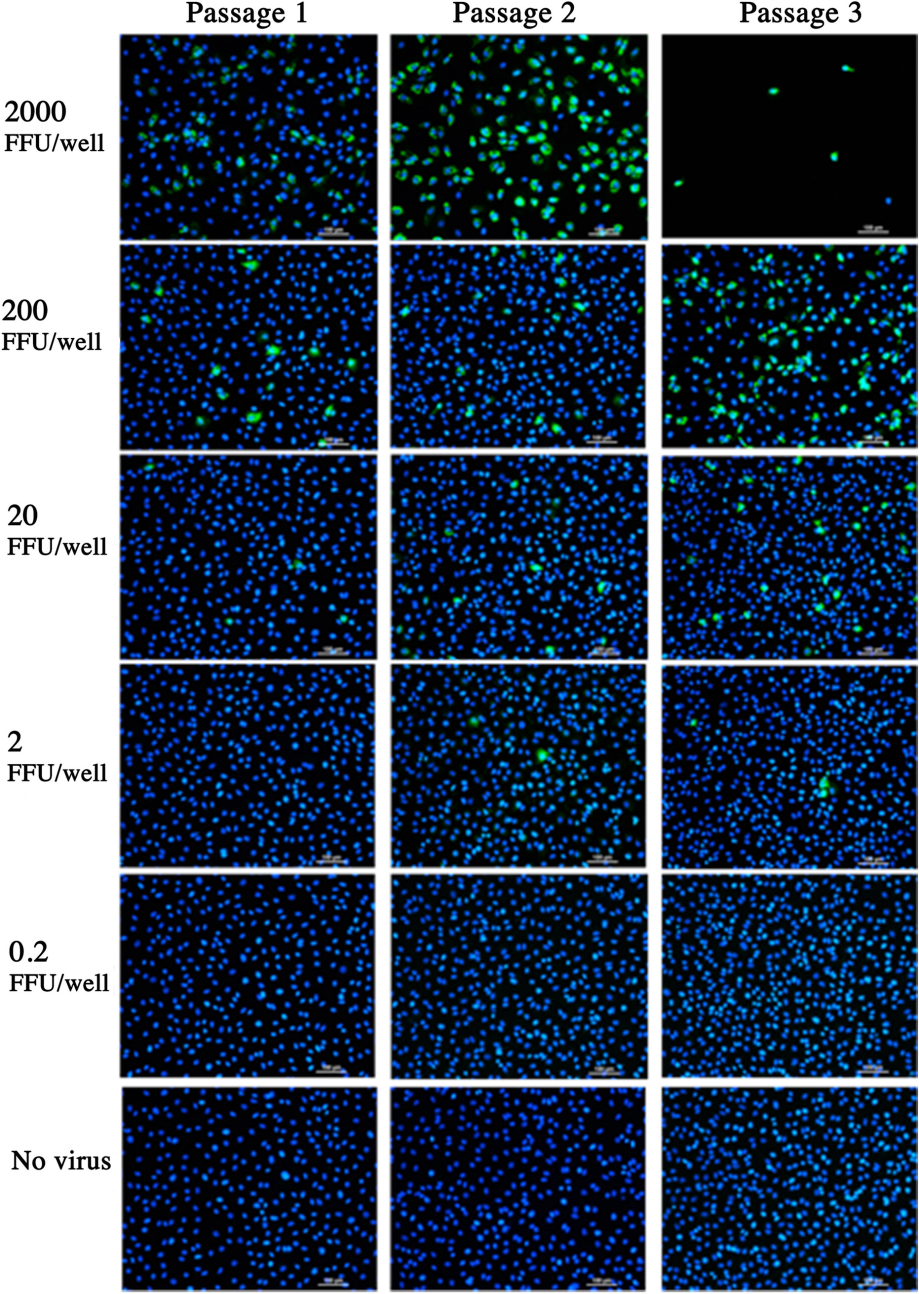
The ability to detect very low FFU of live RV. Monolayers of MA104 cells were incubated for 24h with 0.2 to 2000 FFU live RV. Uninfected MA104 cells were used as a negative control. Culture supernatant was used to infect new MA104 monolayers, and previously infected monolayers were visualised by FFA. DAPI (blue) indicates cell nuclei and FITC (green) indicates RV infection. Stained monolayers were visualised using Nikon TiE inverted fluorescent microscope. Scale bar = 100μm. Images representative of 3 replicates per MOI for each passage.

An irradiation dose of 50 kGy has been widely considered as a mandatory exposure required for the sterilisation of pathogenic agents [37]. Considering that no RV infectivity was detected in samples irradiated with 45 and 50 kGy by FFA, we performed *in vitro* sterility testing to ensure complete virus inactivation. To achieve this, we developed an *in vitro* sterility testing protocol based on serial passages of γ-RV using monolayers of MA104 cells. For this purpose, MA104 monolayers were treated with 4 x 10^5^ FFU-equivalent/well of γ-RV or live RV. Following a 24h incubation period, culture supernatants were collected and used to infect new MA104 cell monolayers. This process was repeated two times, and all previously infected monolayers were stained for RV infection as per standard FFA. As illustrated in Fig 2, in contrast to cell monolayers treated with live RV, monolayers treated with γ-RV preparations irradiated with 50 kGy showed complete lack of detectable infection for all three passages. Furthermore, cell monolayers treated with live RV show considerable cytopathic effect by the third passage, whereas passaging of γ-RV had no negative impact on cell monolayers. In order to demonstrate the sensitivity of the sterility testing and the ability to detect low infectivity levels, MA104 monolayers were treated with serially diluted live RV (2000–0.2 FFU/well) and culture supernatants were collected after an overnight incubation (for 24 h) and used to infect new MA104 cell monolayers. Infection of cell monolayers was clearly detected in monolayers treated with 2000 FFU and the cytopathic effect was evident in passage 3 (Fig 3). Importantly, we detected RV infection in monolayers treated with 2 FFU/well, which illustrate the sensitivity of our sterility testing. Overall, lack of detectable infectivity in monolayers treated with 4 x 10^5^ FFU-equivalent/well of γ-RV illustrates the successful virus inactivation following an exposure to 50 kGy of γ-rays. In addition, materials irradiated with 45 kGy were also considered sterile as no infectivity was detected for all 3 passages (data not shown). Therefore, we proceeded to test the immunogenicity of 50 kGy treated γ-RV preparations in mouse models.

### Immunogenicity of γ-RV

**Fig 4:**
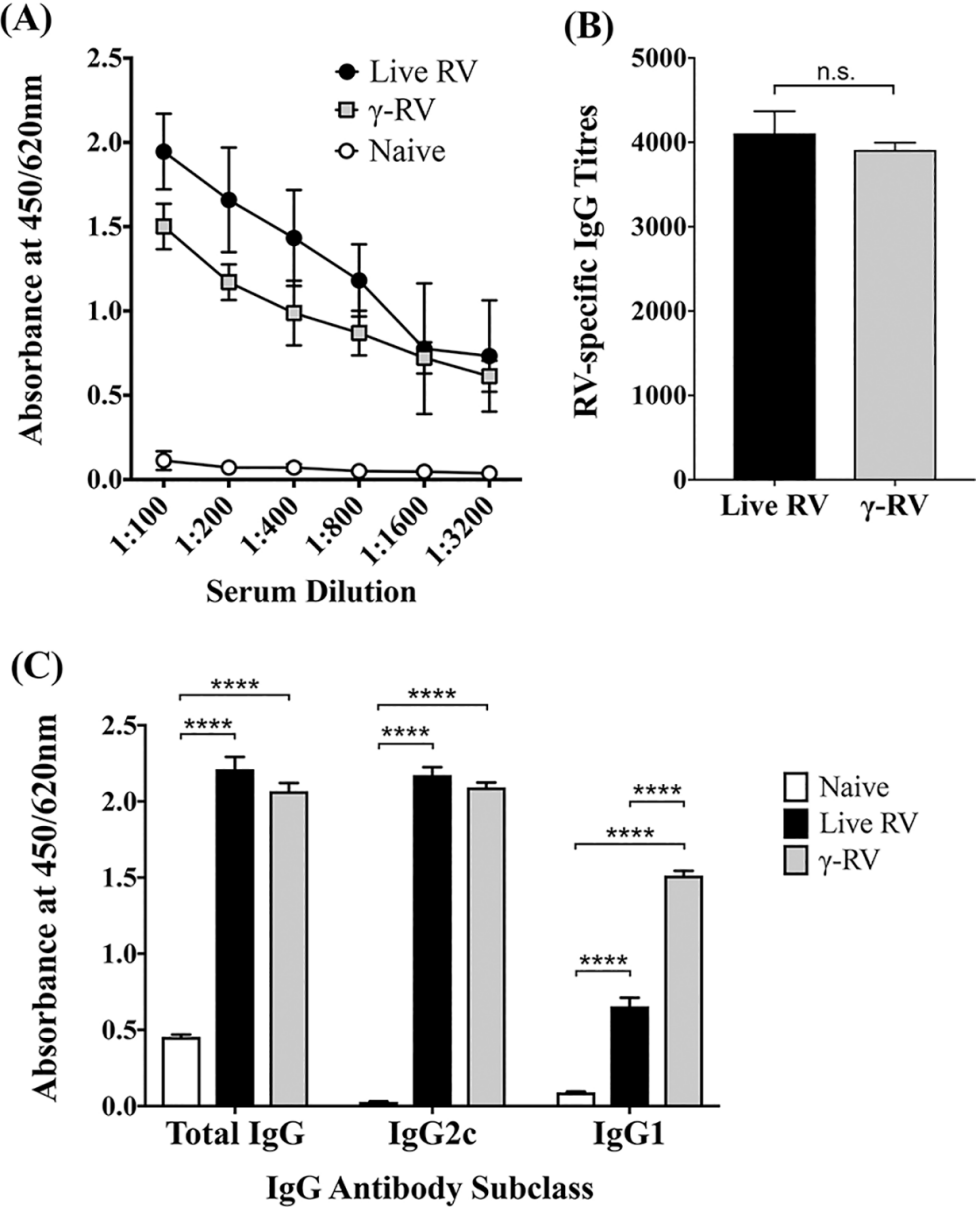
RV-specific antibody responses. Mice were primed twice with live RV or γ-RV, 2 weeks apart. Serum samples harvested on Day 14 post-2^nd^ priming and analysed for RV-specific IgG using ELISA. (A) Serial dilutions of serum samples and absorbance readings at 450/620 nm for total IgG. (B) IgG titres in primed groups calculated relative to cut-off value (dotted line), determined using OD values of serum from control mice. Data presented as mean ± SEM (n = 6), and analysed by unpaired t-test (n.s., not significant) (C) Absorbance at 450/620nm for total IgG, IgG1, and IgG2c in serum at 1:200 dilution by ELISA. Data presented as mean ± SEM (n = 4), analysed by One-Way ANOVA (****, p < 0.0001).

To test proof-of-principle, 8-week-old C57BL/6 mice were injected twice intravenously, with either live RV or γ-RV. PBS-mock treated mice were used as controls. Immune sera was collected 21 days post second injection and tested for RV-specific antibody responses. As shown in Fig 4A, both γ-RV and live RV were able to induce significantly elevated levels of RV-specific IgG in serum compared to PBS-treated controls. End-point titres were also calculated relative to sera from control mice, and we did not detect a statistically significant difference in total IgG induced by live or γ-RV (Fig 4B). Similarly, we detected comparable levels of RV-specific IgG2c in immune sera from both γ-RV and live RV primed mice, with both groups showing a significant increase compared to controls (Fig 4C). Interestingly, we detected a significant increase in IgG1 antibodies in serum samples from γ-RV primed mice compared to sera from mice primed with live RV.

### Neutralising antibody responses induced by γ-RV

**Fig 5:**
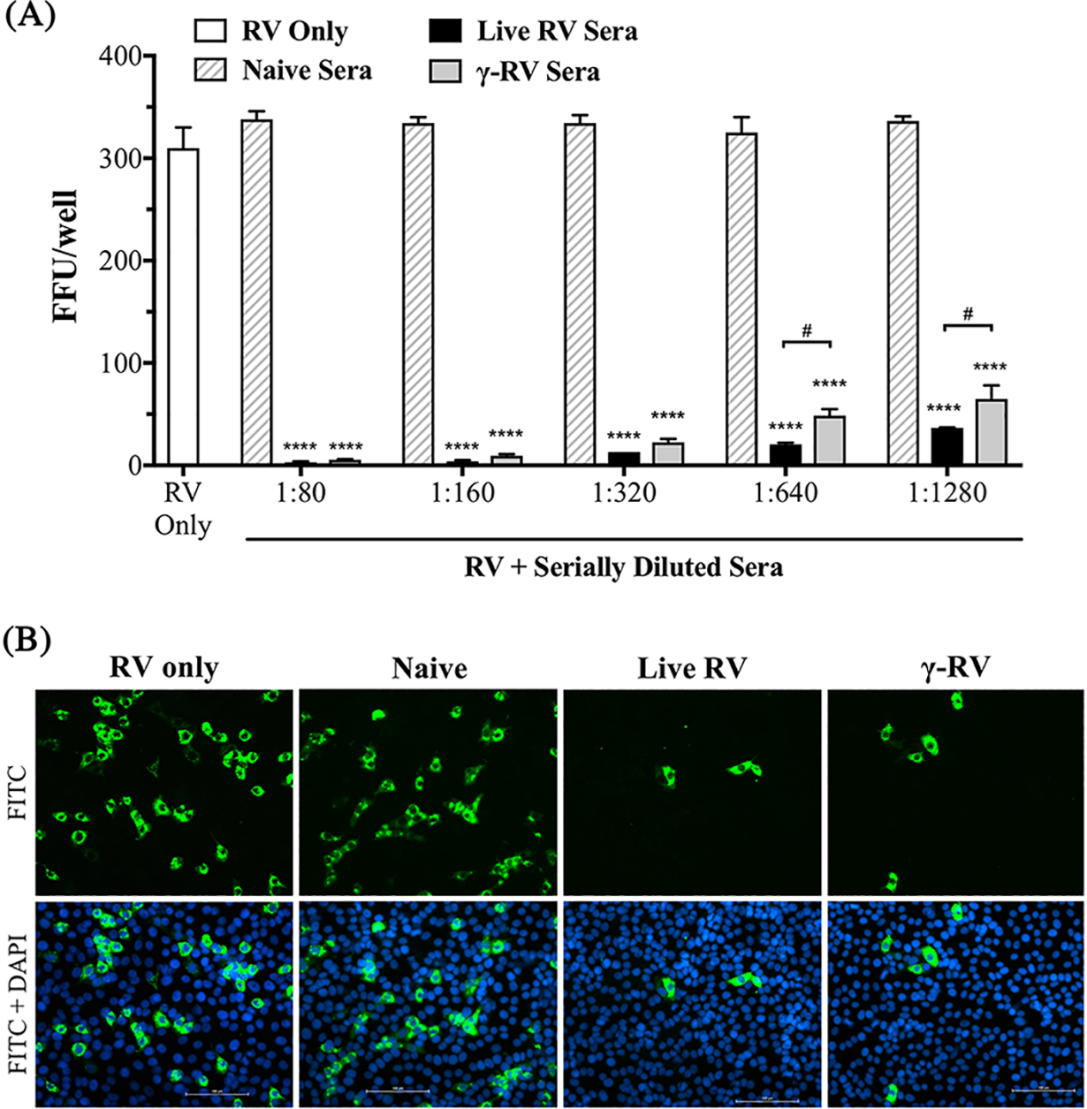
Neutralising antibody responses induced by γ-RV. Mice were primed with live RV or γ-RV twice, 2 weeks apart. Serum samples harvested on Day 21 post-2^nd^ priming, and neutralising ability of immune serum determined by *in vitro* neutralisation assay. (A) FFU/well determined following incubation of MA104 cells with sera treated-RV at MOI 0.005. RV treated with serial dilutions of HI control or immune sera. PBS-treated RV used to indicate the baseline level of infection. Data presented as mean ± SEM (n = 2), and analysed by One-Way ANOVA (****, p < 0.0001 compared to naïve control sera for each dilution. #, p < 0.05, when directly comparing immune sera groups). (B) Representative fluorescence images of RV infection after treatment with live or γ-RV sera at 1:1280 dilution. DAPI channel (blue) indicates cell nuclei, and FITC channel (green) indicates RV infection. Scale bar = 100 μm.

The ability to induce neutralising antibody responses is an essential requirement for highly effective vaccines. Therefore, serum samples from γ-RV vaccinated mice, live RV primed mice, and PBS-treated mice were tested for their ability to neutralise live RV using an *in vitro* neutralisation assay. Heat-inactivated sera samples were serially diluted and mixed with live RV. PBS-treated RV was used as a virus-only control. Monolayers of MA104 cells were then infected with control or sera-treated RV at MOI of 0.005 and quantification of infected cells (FFU/well) following incubation was used to indicate the degree of virus neutralisation. Sera from PBS-treated control mice (naïve control) had no impact on virus infectivity as all tested dilutions show similar FFU counts to that detected for the virus only control. In contrast, treatment of RV with sera from mice primed with either live RV or γ-RV resulted in comparable levels of virus neutralisation across all tested serum dilutions as measured by the significant reduction in detectable FFU/well (Fig 5A). Interestingly, there was also a small difference in the neutralising ability of immune sera from mice primed with live RV versus sera from mice vaccinated with γ-RV. However, this difference was not significant and was only observed with very high dilution factors (1:640 and above). Representative images of infected cells following incubation for 24h with 1:1280 diluted sera-treated RV were also acquired (Fig 5B). Therefore, the ability of immune sera from γ-RV vaccinated mice to neutralise the infectivity of live RV is comparable to that observed for sera from mice primed with live RV.

## Discussion

Despite the high protection rates of live attenuated RV vaccines in developed countries, reduced efficacy has been reported for children living in poor-health conditions [38]. While factors such as malnutrition and diarrhoea may affect the efficacy of oral vaccines in general, the environmental enteropathy, i.e. presence of existing enteric infections, has been reported to be directly associated with reduced efficacy of live attenuated RV vaccines [38, 39]. This has also been observed following oral administration of live attenuated polio vaccine (OPV) [40, 41]. In addition, *in vitro* mixed infection models of RV with astrovirus and enterovirus have been reported to reduce RV replication [42]. While these studies provide possible explanations for the reduced RV vaccine efficacy in developing countries, there is still a clear need for a safer and more effective vaccine strategy for children living in poor-health conditions. We have previously proposed that γ-irradiated pathogens may be used to replace present day vaccines, particularly those associated with complications [33]. In the case of RV, the use of γ-irradiation would negate the risks associated with administration of live virus, whilst retaining high immunogenicity. Additionally, administration of killed virus would overcome the adverse effects of non-related enteric infections on the immunogenicity of current live attenuated vaccines. Therefore, the concept of Sterility Assurance Level (SAL) [43], should be carefully considered. Estimating the radiation dose required to achieve SAL can mathematically be calculated based on the D_10_ value.

Previous studies investigating the possible inactivation of RV using γ-rays have focused on food sterilisation. Consequently, the D_10_ values reported by these studies were calculated for irradiating RV-contaminated food at room temperature [44]. These studies did not address the effect of the complex RV genome structure of RV on the inactivation curve of highly concentrated materials. Importantly, the D_10_ value can be influenced by both the irradiation temperature and media constituents [45]. Therefore, in order to establish the inactivation curve of our RV vaccine, and to illustrate the effect of genome structure on D_10_ value, we prepared RV and SFV (possessing a simple non-segmented ssRNA genome) under similar conditions and tested their sensitivity to γ-radiation. For vaccine purposes however we utilised dry-ice irradiation, as this condition better maintains antigenic epitopes and virus structures even at high irradiation dose [46]. Our data illustrate a log-linear inactivation curve for SFV (Fig 1A), which follows the expected ‘one-hit’ kinetics, indicating that a single virus particle can be inactivated by a single ‘hit’ to the genome. In contrast, we observed a sigmoidal inactivation curve for RV (Fig 1B). It has been reported previously that the presence of alcohols, glycerol, protein and carbohydrates can increase the required γ-irradiation dose for sterilization of virus suspensions [45]. However, both RV and SFV preparations were prepared and inactivated under very similar conditions. Thus, the sigmoidal nature of the RV inactivation curve is not likely to be caused by differences in media components or irradiation conditions. It could however be related to differences in genome structure. The RV genome consists of 11 individual segments of dsRNA. While one hit may destroy one strand of one segment of the genome, the complementary strand remains intact, and could be used for genome replication. In this case, sterilization of a single RV virion requires sufficient damage to both strands of a given genome segment. Thus, data presented in Fig 1B illustrate the effect of genome structure on the susceptibility to inactivation by γ-irradiation. Exposure to 0–30 kGy of γ-rays was associated with a limited drop in virus infectivity, with an exposure to ~15 kGy being required to induce a single log_10_ reduction in virus titre. Following an exposure to 30 kGy however, the RV genome would have been subjected to multiple hits, rendering the preparation more susceptible to an extensive drop in virus titre despite the smaller increases in radiation dose.

Furthermore, the non-linear nature of the inactivation curve meant the D_10_ and SAL values could not be accurately calculated. However, considering that an irradiation dose of 50 kGy has been widely used to inactivate highly infectious materials, we addressed the sterility of 50 kGy irradiated RV using an *in vitro* assay based on serial passages of irradiated materials. Our data clearly illustrate the sterility of 50 kGy γ-RV despite the use of an irradiation dose below the expected SAL, which would exceed 60 kGy based on the inactivation curve and conventional SAL calculations. For clinical applications however, irradiating RV to achieve a SAL (10^−6^ infectious units /mL) suitable for vaccine purposes may not be feasible due to the complicated nature of the RV genome. Instead, it may be possible to use γ-irradiation to inactivate current live attenuated viruses to avoid the need to use an irradiation dose above 50kGy. Achieving SAL for attenuated viruses would not be necessary given that they are currently approved for clinical application. Considering the clinical complications associated with live attenuated RV vaccines, inactivation by irradiation would enhance safety and ensure vaccine efficacy. Therefore, we tested the immunogenicity of 50 kGy γ-RV in mice. Our data clearly illustrate the ability of γ-RV to trigger strong RV-specific humoral immune responses comparable to those induced by live RV, particularly the induction of high levels of IgG2c. Previous studies have illustrated the ability of heat inactivated RV to induce neutralising Ab responses in animals [47, 48]. In contrast to these studies, our data show high immunogenicity of γ-RV without using adjuvant. It has been reported previously that analysis of the ratio of IgG2c: IgG1 isotypes can indicate the induction of Th1 or Th2 type immune responses [49]. Measurement of antibody isotypes in immune sera from primed mice showed that γ-RV induced higher IgG2c levels compared to IgG1, suggesting a bias towards a type-1 immune response. This is consistent with higher IgG2c levels than IgG1 detected following vaccination with other γ-irradiated viruses, such as γ-SFV [36]. This outcome is commonly desired within vaccine development due to the competent functions of IgG2c during antigen clearance [50, 51]. Interestingly, despite the higher IgG2c levels compared to IgG1 detected following priming with γ-RV and live RV, our data show higher level of IgG1 in serum samples from γ-RV vaccinated mice, in comparison to sera from mice priming with live RV. This may be related to differences in cytokines induced in response to killed versus live RV, which would require further investigations. Nonetheless, both live and γ-RV induced comparable levels of total IgG and IgG2c. In addition, our data regarding virus neutralization illustrate that γ-RV elicits humoral responses of comparable strength to those induced by live RV. This highlights the potency of γ-RV in inducing antibody responses without using adjuvant.

Overall, we investigated the possibility of using γ-irradiation to inactivate RV for vaccine purposes. While our data indicate that a dose considerably higher than 50 kGy would be required to achieve the internationally accepted SAL for irradiated RV, *in vitro* testing confirmed the sterility of 50 kGy γ-RV. In addition, we have illustrated that γ-RV induces strong neutralising humoral immune responses comparable to those induced by live RV. Gamma-irradiated preparations can be administered in considerably higher doses than their live viral counterparts, allowing for a dramatic increase in the total antigen dose without causing infection or the associated clinical complications. Therefore, γ-irradiation of current live attenuated RV vaccines could be utilised to overcome their reduced efficacy, and enhance safety without comprising immunogenicity.
